# New Cyt-like δ-endotoxins from *Dickeya dadantii*: structure and aphicidal activity

**DOI:** 10.1038/srep08791

**Published:** 2015-03-05

**Authors:** Karine Loth, Denis Costechareyre, Géraldine Effantin, Yvan Rahbé, Guy Condemine, Céline Landon, Pedro da Silva

**Affiliations:** 1 INSA-Lyon, Villeurbanne F-69621, France; 2 CNRS, UMR5240 MAP, Microbiologie Adaptation et Pathogénie, F-69622, France; 3 Université Claude Bernard Lyon 1, F-69622, France; 4 Université de Lyon, F-69000 Lyon, France; 5 Centre de Biophysique Moléculaire, CNRS UPR 4301, Université d'Orléans, Orléans, F-45071, France; 6 INRA, UMR203 BF2I, Biologie Fonctionnelle Insecte et Interaction, F-69621, France

## Abstract

In the track of new biopesticides, four genes namely *cytA*, *cytB*, *cytC* and *cytD* encoding proteins homologous to *Bacillus thuringiensis* (*Bt*) Cyt toxins have been identified in the plant pathogenic bacteria *Dickeya dadantii* genome. Here we show that three Cyt-like δ-endotoxins from *D. dadantii* (CytA, CytB and CytC) are toxic to the pathogen of the pea aphid *Acyrthosiphon pisum* in terms of both mortality and growth rate. The phylogenetic analysis of the comprehensive set of Cyt toxins available in genomic databases shows that the whole family is of limited taxonomic occurrence, though in quite diverse microbial taxa. From a structure-function perspective the 3D structure of CytC and its backbone dynamics in solution have been determined by NMR. CytC adopts a cytolysin fold, structurally classified as a Cyt2-like protein. Moreover, the identification of a putative lipid binding pocket in CytC structure, which has been probably maintained in most members of the Cyt-toxin family, could support the importance of this lipid binding cavity for the mechanism of action of the whole family. This integrative approach provided significant insights into the evolutionary and functional history of *D. dadantii* Cyt toxins, which appears to be interesting leads for biopesticides.

Weeds, pathogens and animal pests are potentially responsible for huge economic losses in agricultural production, and about 20% of these losses are due to animal pests^1^. Among insects, aphids (Hemiptera: Aphidoidea) are one of the most injuring taxa for agricultural plants. They are difficult to control due to their specialized feeding mechanism and unusual reproductive biology^2^,^3^. As a result, the management of aphid populations is quite challenging. Until now, most aphid pest control strategies rely on the use of specific sets of systemic chemical pesticides. But the extensive use of these pesticides had led to resistance to insecticides in several aphid species^4^,^5^, and cause significant environmental damage by targeting different guilds of beneficial insects (predators, parasitoids, and pollinators)^6^,^7^. Therefore, it is highly desirable to develop biopesticides with low non-target effects. A substitute to current chemical pesticides is plant bioengineering; in order to be more selective to target pests, plants can be genetically modified to express insecticidal biomolecules within specific tissues^8^. Nevertheless, as happened with conventional pesticides, plant bioengineering has also led to some field insect resistance^9^. Hence, crops with more than one defensive protein, each with a different mechanism of action, have been proposed to delay insect resistance (gene pyramiding). However, very few genetically modified plants have yet been developed with resistance to sap-sucking insects, and none is used commercially^10^,^11^,^12^. Therefore it seems essential to further develop suitable biopesticides, which could turn into candidate genes for the development of aphid-tolerant plants.

In the track of such biopesticides, bacterial toxins did provide paradigmatic solutions, such as the crystal toxins encoded in plasmids of the soil bacterium *Bacillus thuringiensis* (*Bt*), which provided a vast diversity of Cry-like toxins for plant protection. Recently however, *Bt*-related toxins were found in genomes outside its original Gram+ bacterium: four genes namely *cytA*, *cytB*, *cytC* and *cytD*, encoding proteins homologous to *Bacillus thuringiensis* (*Bt*) Cyt toxins have been identified in the *Dickeya dadantii* (formerly *Erwinia chrysanthemi*) genome^13^. The low GC content of these genes in *D. dadantii* suggests horizontal transfer from a GC-poor Gram+ bacterium^14^. *Bt* Cyt toxins are produced in parasporal crystals during sporulation, together with the better-known Cry toxins^15^. So far three families of Cyt proteins, Cyt1, Cyt2 and Cyt3 have been identified (http://www.btnomenclature.info). They are active by ingestion and, after proteolytic maturation, they perforate the membrane of insect intestinal cells through a presumed receptor-independent pathway, by attaching non-specifically to phospholipids^16^. The mechanism of action of *Bt* Cyt toxins is not fully understood yet. The plant pathogenic bacteria *D. dadantii* was also shown to be a pathogen of the pea aphid *Acyrthosiphon pisum*^17^. Artificial infection of *A. pisum* by *D. dadantii*, *via* an oral route, provokes the death of the insect in about four days. When delivered *per os*, the reduced virulence of a *D*. *dadantii* strain deleted for all four *cyt* genes suggests that these proteins are involved in its pathogenicity to insect. When the mutant is delivered by injection into the hemocoel, the virulence is not reduced, evoking an intestinal cell target for the *Dickeya* Cyt proteins^17^.

To better understand the role of *D. dadantii* Cyt toxins in its pathogenicity to insect, we defined the following strategy for the present study: i) the four recombinant proteins were purified and were used for toxicity bioassays against the pea aphid *A. pisum*; ii) a phylogenetic analysis was performed to investigate the evolutionary and functional relationships within the whole Cyt-like protein family, iii) from a structure-function perspective, the CytC 3D structure and its dynamics in solution have been determined by NMR.

This integrative approach provided significant insights into the evolutionary history of *D. dadantii* Cyt toxins, which appear to be interesting leads for biopesticides, in parallel to the screens still performed within the *Bacillus thuringiensis* species.

## Results

### Protein purification and protein compliance

To investigate the biological activity of the four *D. dadantii* Cyt proteins, we tried to produce these proteins as GST-fusion proteins expressed in *E. coli* cells and purify them. Some difficulties were encountered in producing some of these proteins in our bacterial system due to their low solubility. We could not produce CytD protein because it was highly insoluble and formed inclusion bodies. Low production levels were obtained for CytA and CytB (less than 40 μg for 1 L of culture). The quantity purified was however sufficient to perform some biological tests. The production level of the CytC protein was far better (around 0.4 mg for 1 L of bacterial culture). SDS-PAGE analyses showed that the CytA, CytB, CytC proteins were of high purity, and with the expected size (Figure S1). Due to its production level, CytC was selected for further NMR structural studies. The purified protein recovery was independent of isotopic feeding conditions.

### Biological assays

Different biological assays were performed to assess, and compare the functionalities of the produced proteins with those of the *B. thuringiensis* proteins.

#### Insect bioassays

**Figure 1:**
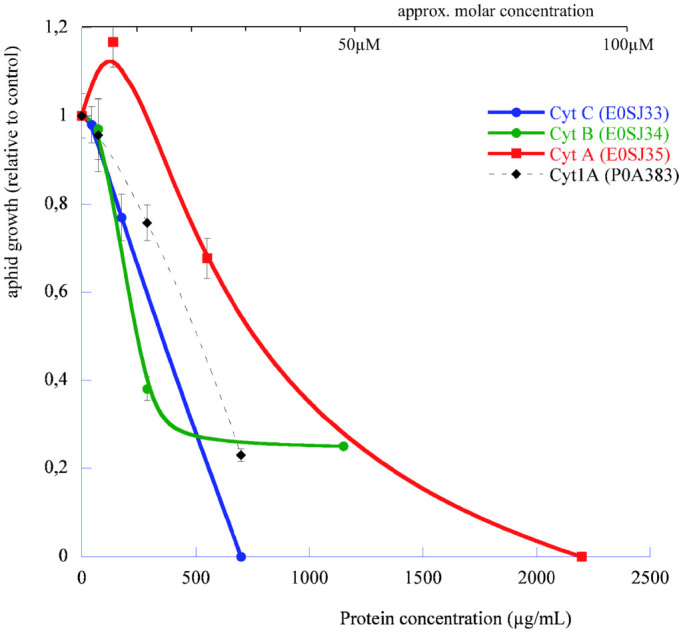
Growth inhibition of the pea aphid by *Dickeya dadantii* Cyt toxins. Aphid weight (normalized to mean of control group) was measured at the end of the toxicity assay, at which control aphids were adult. Bars represent standard error of means. Protein codes are Uniprot IDs of the tested toxins. Cyt1A was tested in an independent assay (experiment 3) from the *Dda*-Cyt toxins (experiment 2).

Figure 1 summarize the insect toxicity bioassays performed by ingestion with the pea aphid and four available Cyt-like proteins: one from *Bacillus thuringiensis* subs. *israelensis*, a typical solubilized dipteran-specific toxin Cyt1A, and the three available *D. dadantii* toxins (CytA, CytB and CytC).

Survival analysis of the associated data showed that all tested proteins induced weak but significant acute mortality on pea aphid nymphs between 250 and 1000 μg/mL, with the following quantitative trends: Cyt1A (Uniprot ID: P0A383) intoxication at 1000 (resp. 250 μg/mL) resulted in LT50 (Lethal Time 50%, in days, and confidence interval under a Weibul fit) of 3.24 [2.11–4.98] and 12.8 [10.3–16.0] respectively. In the parallel assay, *D.dadantii* CytC (Uniprot ID: E0SJ33) at 1000 (resp. 250 μg/mL) resulted in LT50 of 10.1 [5.9–17.3] and 9.6 [8.4–11.0] respectively. In the same assay at 500 (resp. 250 μg/mL), Cyt B (Uniprot ID: E0SJ34) resulted in LT50 of 5.1 [3.7–7.1] and 8.1 [7.3–9.1] respectively. Due to toxin availability and stability issues with the recombinant CytB and CytA proteins (not CytC), assays were not all performed with the same concentration range or in a single bioassay batch. In an independent experiment, CytA (Uniprot ID: E0SJ35) resulted in LT50 of 2.28 [1.84–2.82], 15.7 [8–31] and 22 [11–43] respectively at 1000, 500 and 125 μg/mL.

Overall, the different bioassays showed that all three tested Cyt toxins displayed aphicidal activities somewhat similar to that of Cyt1A^18^, with a strong growth-reducing effects (Figure 1) in the 250–500 μg/mL range. Comparing all experiments resulted in a global ranking of toxicity as follows: *Dda*-CytB > *Dda*-CytC ≈ *Bth*-Cyt1A ≈ *Dda*-CytA. More precisely, LT50 of Cyt B is the smallest at active dose (*e.g.* 8.1 days at 250 μg/mL). The most striking phenotypical effect, previously observed with *B. thuringiensis* Cyt1A^18^ as a severe growth impairment of the pea aphid at moderate doses, was reproduced with *D. dadantii* Cyt endotoxins with more than 60% growth inhibition, CytB being the most active at low active dose (Figure 1).

#### Hemolysis assays

No hemolytic activity was observed for the CytA (up to 120 μg/mL, 5.4 μM), CytB (up to 30 μg/mL, 1.3 μM) and CytC (up to 1000 μg/mL, 45 μM) proteins. This is to compare to the low nanomolar range (44 nM) published for the hemolytic activity of Cyt1Aa^19^, reproduced in our assays as a full hemolysis for trypsin-activated Cyt1A toxin at less than 1 μM. Trypsin did not activate CytC in our hemolysis assays.

### Phylogeny of the Cyt-like family

**Figure 2:**
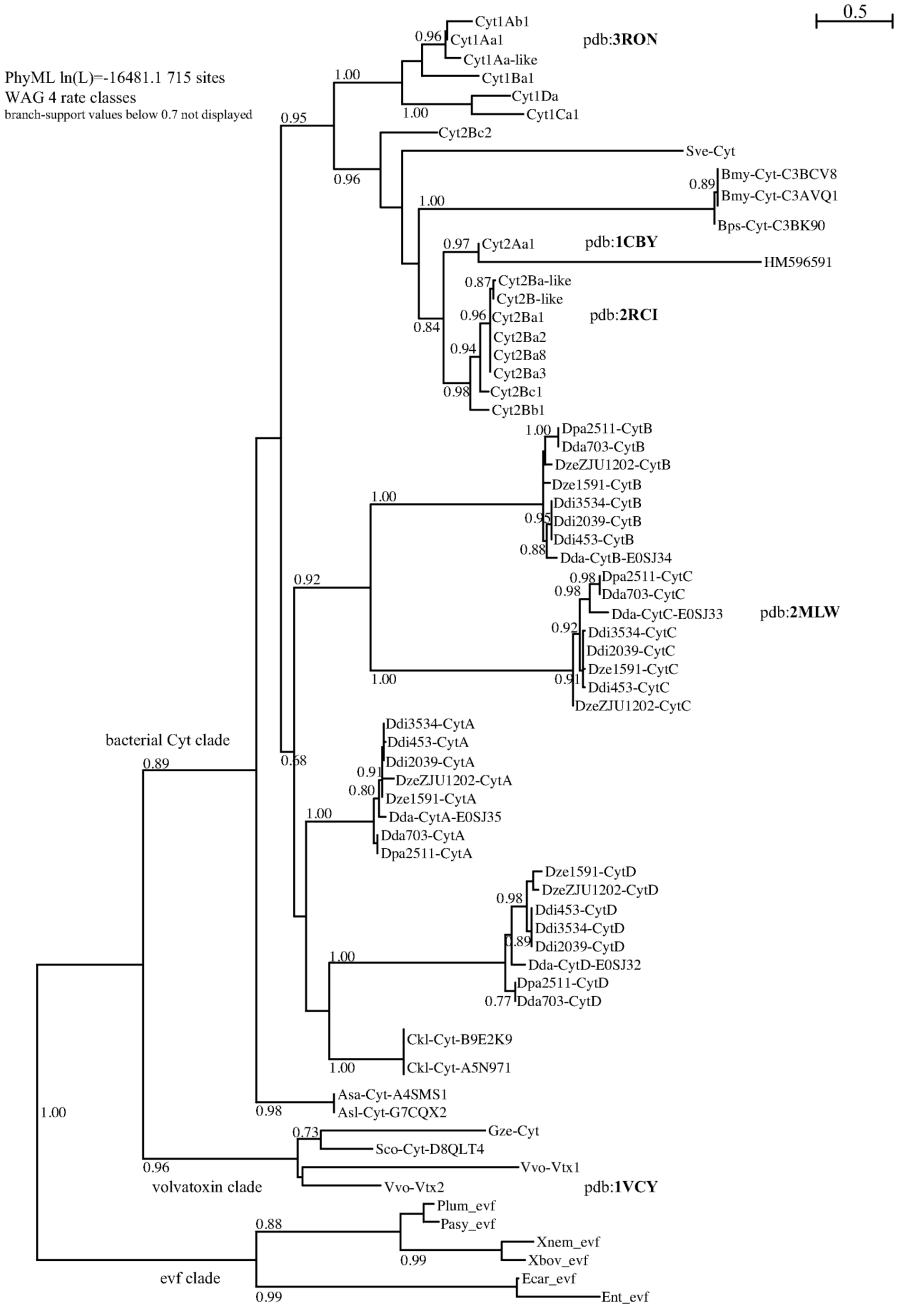
Unrooted phylogenetic tree of all non-redudant cyt-homologous toxins that were retrieved from Genbank, Uniprot and specific genome databases. Phylogenetic reconstruction, on aligned protein sequences followed a PhyML method^50^ with a WAG 4-rate class model. The early three separate clades are labeled *evf*, *volvatoxin* and (other) *bacterial cyt* clades. Labels are built to allow both easy reading and non-ambiguous peptide identification: *Bacillus thuringiensis* toxins are not preceded by the species suffix (otherwise: three letter suffix-id, Gsp for *Genus species*), follow the Crickmore holotype classification, and labels include the Uniprot accession number for non-*Bt* species.

**Figure 3:**
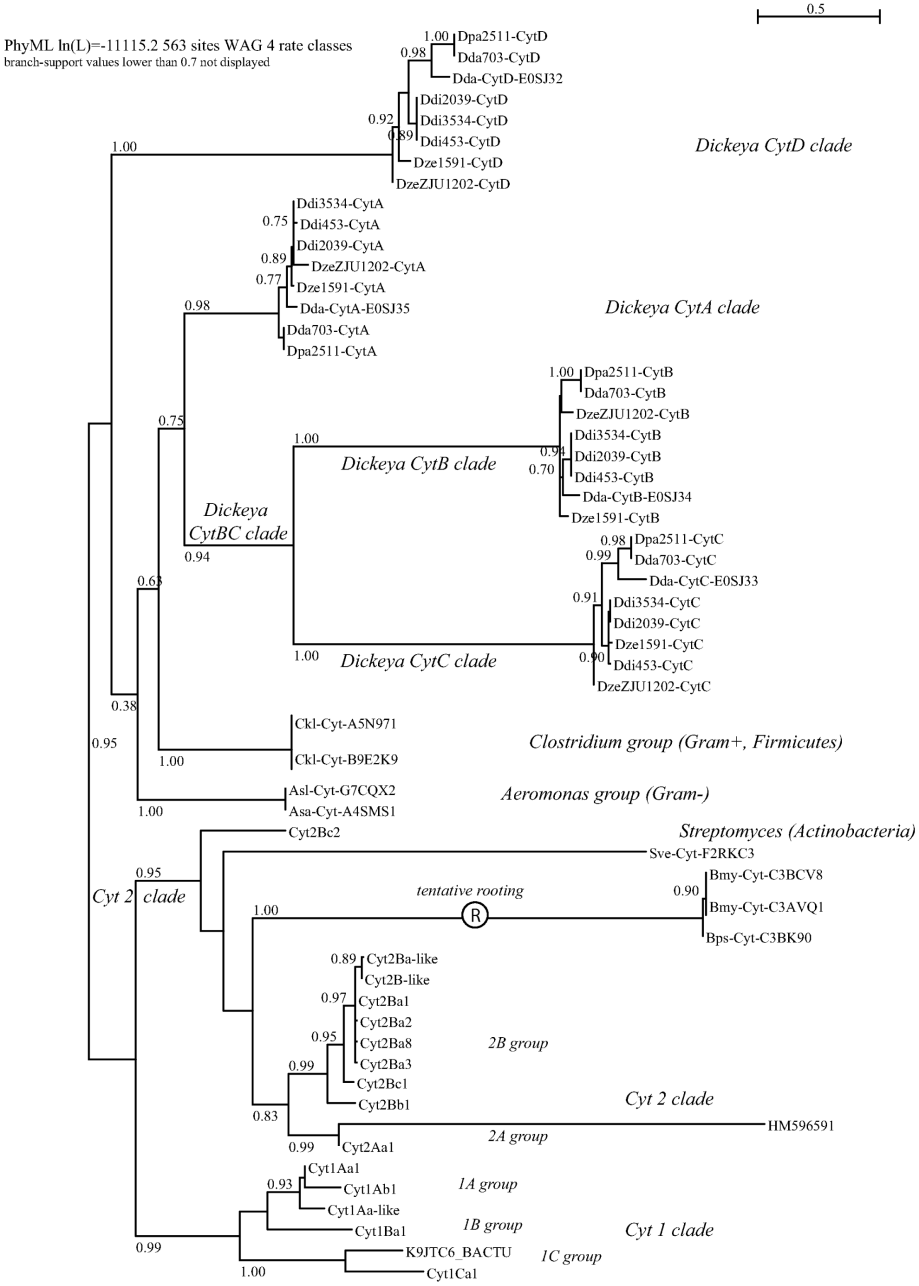
Phylogenetic tree of all *bacterial cyt* toxins; tree is unrooted but tentative rooting at longest branch is proposed ®. Method and labels as in figure 2.

We aimed at identifying the whole set of proteins homologous to the *D. dadantii* and *B. thuringiensis* Cyt proteins. The Blast-based homology search on Uniprot and Genbank peptide databases retrieved 70 non-redundant sequences, which were aligned and analyzed through a maximum-likelihood phylogeny, presented as an unrooted tree in Figure 2 (450 patterns out of 715 sites, 277 non-polymorphic sites -39%-). Deep branches identified the three independent clades that were only recently grouped within a single family through structural alignments^20^,^21^; we named these the volvatoxin clade, the Evf clade and the bacterial Cyt clade. All these proteins share the cytolysin fold. In order to investigate the bacterial Cyt clade with more accuracy, we excluded the two other clades to reduce saturation and re-performed the analysis (Figure 3; 404 patterns out of 563 sites, 163 sites -29%- found without polymorphism). The canonical *Bacillus* (Firmicute) Cyt toxins were grouped in two well resolved clusters, the Cyt 1 and Cyt 2/3 clades; the only taxonomical outlier in the latter cluster was the *Streptomyces* (Actinobacteria) toxin. The remaining clusters were not resolved basally, which resulted in the unsolved positioning of the *Clostridium* (Firmicute) group as related to the *Dickeya*/*Aeromonas* groups (Proteobacteria). The *D. dadantii* toxins derive from lateral gene transfers as testified by their still-evolving GC% within large genomic pathogenicity clusters^13^,^14^. The recovery of the same 4-*cyt* gene toxin cluster in the eight *Dickeya* genomes shows that this linkage predates the speciation events within this bacterial group. Within the Cyt toxins *sensu stricto* (*i.e.* excluding Evf and Volvatoxin clades), the most probable scenario seems to be an invention/diversification of the toxins within the Firmicutes (*Bacillus*/*Clostridium*) followed by three episodes of horizontal gene transfers towards independent and distant bacterial clades (*Dickeya*, *Aeromonas*, *Streptomyces*).

### Cyt toxin family nomenclature

Figures 2 and 3 represent to our knowledge the most comprehensive Cyt trees to-date, and contain phylogeny-based nomenclatural updates for the Cyt toxin family, as defined and classified previously^22^,^23^. We propose that, independent on gene namings, proteins are short-named “only” « Cyt[A-Z] » and described as « type-[N] cytolytic delta-endotoxin » (as most-often encountered in Uniprot and exemplified by accession P0A382 for example). When clusters of paralogs are found in single genomes, such as in *Dickeya* sp., the [A–Z] suffix should ideally be attributed only after adequate orthology assignment is performed within the given clade.

### CytC solution Structure

**Figure 4:**
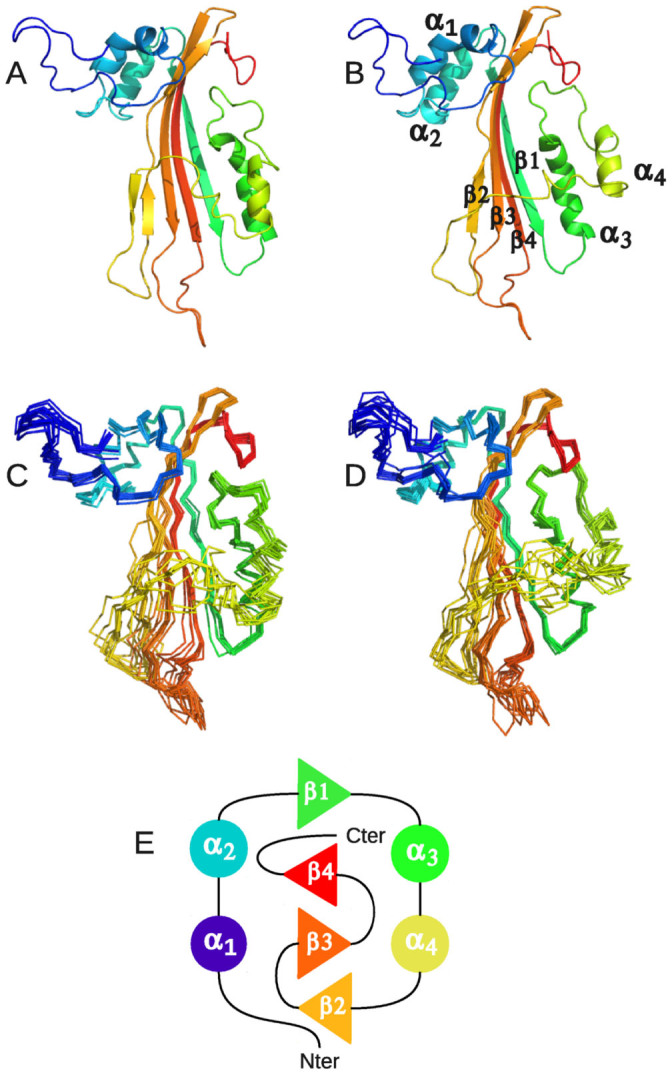
Ribbon representation of model 1 (A) and 11 (B) of the NMR ensemble of CytC (2MLW.pdb). Overlay of C^α^ traces of models 1 to 10 (C) and 11 to 20 (D). (E) Topology diagram of CytC. Helices and strands are represented by circles and triangles respectively. Secondary structure elements are colored in rainbow starting from blue to red.

CytC structure was determined by NMR on the protein labeled with ^15^N and ^13^C. Chemical shift assignments were obtained for 95% of the backbone and 75% of the proton side-chains (BMRB code 19834). The NMR 3D structure of CytC (PDB entry 2MLW) was determined using NOE distances, dihedral angles and hydrogen bonds (Table 1). As a member of the Cyt protein family, CytC has a cytolysin fold, i.e. a single domain of α/β architecture consisting of a β-sheet surrounded by two α-helical layers (Figure 4). The sheet consists of 4 main anti-parallel β-strands having a modified Greek key topology composed of β2 (T137-G149), β3 (G153-T166) and β4 (L182-V193) connected by a longer link to β1 (A63-K74), which is adjacent to the first strand β2. The sheet is flanked by two α-helical layers: α1 (Q29H39) and α2 (K51-A58) on one side, and α3 (L79-E93) and α4 (N106-F112) on the other (Figure 4). Some of the NMR models contain a supplementary very short β-strand (F124-N127), antiparallel to β2.

**Figure 5:**
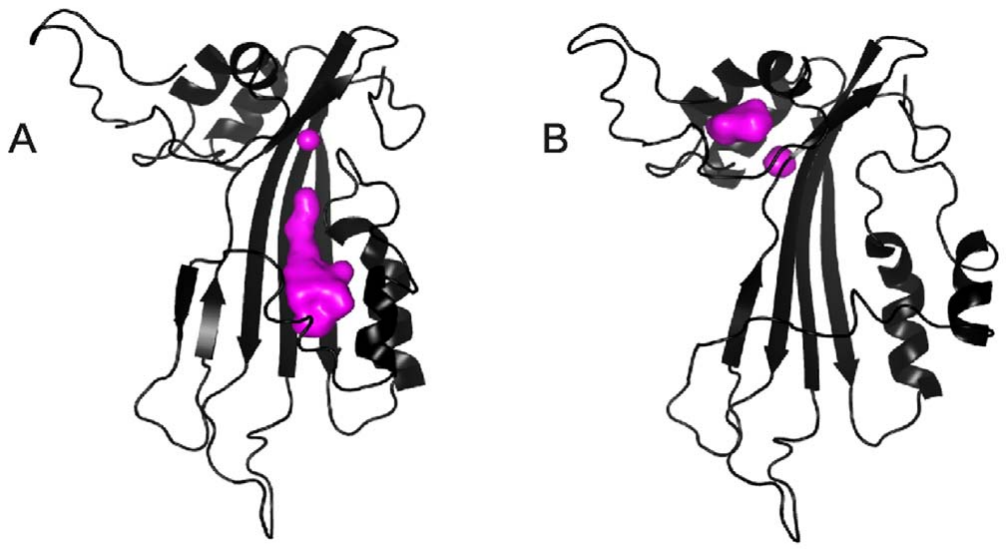
Cavity of CytC protein in the two models of NMR structures; the cavity is in purple. (A) Models 1 to 10 and (B) Models 11 to 20.

Interestingly, the NMR ensemble of CytC structures contains two distinct and equally populated conformations of the protein in solution, which are in agreement with our experimental data (Figure 4 A and B). These two conformations differ principally by the position and orientation of α4 with respect to the β-sheet defining an ensemble of “closed” (models 1 to 10) or “opened” (models 11 to 20) conformations. The “closed” conformation, in which α4 is closer to the β-sheet, exhibits a quite large hydrophobic pocket (~1000 Å^3^, detected by Pymol (32) and measured by Castp (34)), defined between the β-sheet and α3 and α4 in which a hydrophobic ligand might interact with the protein. This pocket is absent in the “opened” conformation, allowing residues defining this pocket in the first conformation to be accessible to the solvent and/or a ligand (Figure 5). However, the NMR spectra used for the backbone and side-chains resonances assignment do not contain any peaks suggesting that the protein is present in two distinct forms in solution. This lead us to conclude that the two conformations are in a fast exchange regime if both exist.

The^15^N-HSQC spectrum of CytC recorded at 600 MHz showed good dispersion and signal to noise ratio for only 127 backbone N-H cross peaks (62.6% of the protein residues) to obtain quantitative R_1_, R_2_ and NOE. ^15^N R_1_, R_2_ and NOE values are constant all along the protein sequence with values of 0.90 ± 0.08 s^−1^, 16.45 ± 2.25 s^−1^ and 0.91 ± 0.12 s^−1^ respectively. However, residues A128 to V136, comprised between β2 (T137-G149) and β3 (G153-T166) exhibit lower values for the ^15^N heteronuclear NOE and transverse relaxation rate than the rest of the protein indicating enhanced mobility.

For rigid protein molecules, in the limit of slow molecular motion (τ_c_ ≫ 0.5 ns) and high magnetic field, a closed-form solution for τ_c_ as a function of the ratio of the longitudinal (T_1_) and transverse (T_2_) ^15^N relaxation times exists:
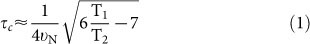
where υ_N_ is the ^15^N resonance frequency (in Hz). This equation 1 is derived from Eq. 8 from Kay *et al*.^24^ by considering only J(0) and J(ω_N_) spectral density terms and neglecting higher frequency terms. Using this equation, CytC τ_c_ was estimated to be 13 ns. By comparing this to a table of rotational correlation time values for known proteins, we can conclude that our NMR sample of CytC has a molecular weight around 22 kDa and that it is a monomer.

## Discussion

The core of this study was to investigate the role in pathogenesis of the homologous Cyt-proteins that are present in *D. dadantii* genome. Consequently, we focused on the following key question: do CytA, CytB, CytC and Cyt D proteins present insecticidal activities? To address this question, toxicity bioassays against the pea aphid were performed with recombinant form of the protein expressed in *E. coli*.

The biological activity data clearly show that the *Dda*-Cyt genes encode insecticidal proteins active against the pea aphid, and that this activity was at least as potent as that of its parent *Bth*-Cyt1A protein^18^. Protein CytD could not be produced in the expression system used. However, its persistent outward positioning and long-branching in the phylogeny may reflect a structural peculiarity or a nascent pseudogenisation process. The activity range of CytA, CytB and CytC between 125 and 500 μg/mL was similar to that of a wild-type Cyt2A on the same insect target^25^, but displayed less apparent toxicity than the original mosquitocidal activity (LC50s around 1–10 μg/mL^26^, although comparing a soluble food (aphid) and a particulate food/living media (mosquito larva) is not a trivial process. The most striking toxicological phenotype of the Cyt toxins is the severe growth-stunting effect (Figure 1), resulting in surviving individuals being three to four times smaller than normal. This is a clear indication of lack of physiological adaptation of the gut cell to the toxin action. Impairment of the microvillar structure of aphid enterocytes was shown to be the most visible cellular phenotype of Cyt2A intoxication in an aphid^25^, consistent with the membrane-related mode of action of Cyt toxins, either through a detergent or a pore-forming mechanism^20^. Noteworthy, no synergism between Cyt1A and any of the tested Cry toxins^18^ was observed with the pea aphid (Porcar and Rahbé, unpublished), in contrast to the situation described with Cry11 on mosquito^26^,^27^. All these results indicated that the Cyt toxins of *Dickeya dadantii* were probably evolved outside their original *Bt* ecosystem to perform (alone) their pore-forming function in the digestive tract of target insects. This is consistent with the previous finding of i) a virulence function when the bacteria was ingested but not by injection, as measured by differential virulence of *wt* and Δ*cyt* *D. dadantii* strains^17^, and ii) gut-restricted expression of the *cyt* operon^28^. One series of interesting and selective features of the *Dda*-Cyt toxins is i) their lack of hemolytic activity, as compared to the parent *Bt*-Cyt toxins^19^,^29^, ii) their N-terminal shortening, and hence absence of need for proteolytic activation, which was experimentally checked in the present work for hemolytic activity, and iii) their ability to be expressed alone in a bacterial cytoplasm, whereas the *Bt*-Cyt toxins sometimes display bacterial cell toxicity^30^ and need a helper gene to be expressed in a standard intracellular bacterial context^31^. An analysis of Cyt1Aa mutants has shown that the mutations V122E and V126E affected strongly the oligomerization and haemolytic properties of the proteins^32^. Interestingly, the homologous residues in CytC, the least prone to aggregation of the *D. dadantii* toxins, are Q89 and E93, which could explain the properties of CytC. However, other changes should explain the absence of haemolytic activity of CytA and B since a leucine is found at the position corresponding to V122 and a valine is conserved at the position corresponding to V126 (Figure 5). It seems unlikely to us that the distinctive properties of the *D. dadantii* Cyt properties could have evolved without appropriate selective pressures. The first trait (hemolysis) should involve target organism specificity, while the other trait (bacterial compatibility) should involve adaptation to recipient host cytoplasmic expression. Both are meaningful but deserve further experimental analysis.

The phylogenetic analysis of the comprehensive set of Cyt toxins available in genomic databases shows that the whole family is of limited taxonomic occurrence, though in quite diverse microbial taxa. Together with some information on their genomic context (*e.g.* positioning in pathogenicity islands and GC content, for the *Dickeya dadantii* toxins), this is indicative of a diversification in a somehow restricted and specialized set of species (e.g. Firmicutes/*Bacillus* species), followed by rare recurring events of horizontal gene transfers (HGT) and fixation in other lineages (such as in some fungal –*Volvariella*, *Giberella*– or γ-proteobacterial –*Dickeya*, *Aeromonas*– species). A possible positioning of a root in the presented tree was attempted for such a scenario (Figure 3): the longest identified branch between the Cyt2 clade and the other *Bacillus* taxa could be this basal point, leading to a set of three successive events of HGT into unrelated taxa (*Streptomyces*, *Aeromonas* and *Dickeya*). *Clostridium* is a much closer taxon to *Bacillus*, and its positioning in the tree is less clear. It is interesting to note that a related group of filamentous bacteria, *Arthromitus* (Firmicutes, Clostridiales), have long been described in association to arthropods and insects, and showed both fossil and extant records of association with insects^33^. Whether a new set of toxin folds, modules and assemblages (both the Cyt and Cry toxins contain specific pore-forming folds) arose from such a position in the bacterial tree may be seen as a challenging hypothesis, not properly studied yet even in the most comprehensive genomic analyses of the *Bacillus* sp genomes published to date, which show that both *cry* and *cyt* genes occur exclusively on plasmids in the 45 *Bacillus* genomes explored^34^. At the other end of the tree, the reconstructed topology (Figure 3) shows clearly that the *Dickeya* toxin cluster evolved in a multistep process involving a double tandem-duplication step, giving birth to the CytBC clade (proteins shorter than the two other groups, ≈200 residues vs ≈220 residues for CytA and CytD).

**Figure 6:**
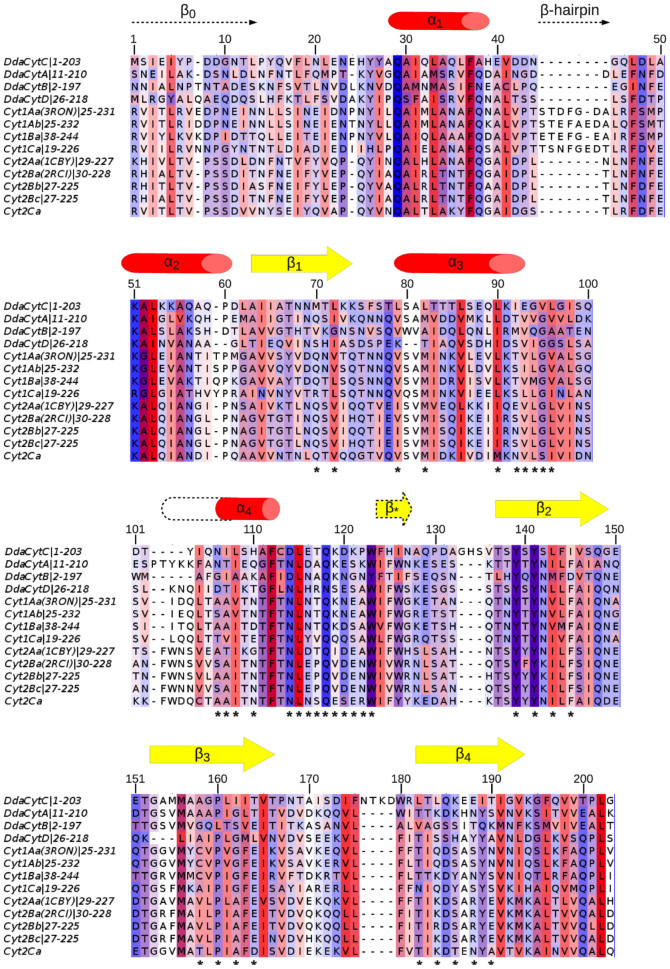
Sequence alignment of Cyt from *D. dadantii*, Cyt1 and Cyt2 family members. CytC secondary elements are labeled above the corresponding sequence. Helices are represented by red cylinder and strands by yellow arrows. Numbers refer to the CytC sequence. The residues are colored by their hydrophobicity properties from red (hydrophobic) to blue (hydrophilic) and by conservation. The conserved residues forming the cavity are marked by black asterisks.

**Figure 7:**
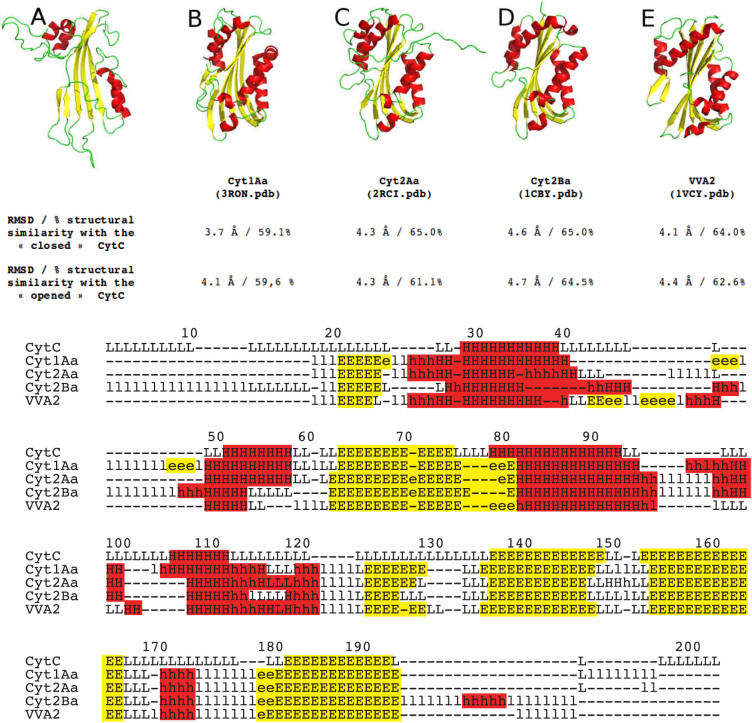
Ribbon representation of CytC (A), Cyt1Aa (B), Cyt2Aa (C), Cyt2Ba (D), and VVA2 (E). Helices are represented in red and β-strands in yellow. The percentage of similarity and the RMSD of two “closed” and “opened” CytC conformations with other Cyt toxins (Cyt1Aa (B), Cyt2Aa (C), Cyt2Ba (D), and VVA2 (E) are indicated. The second part shows the secondary structure alignment (H/h: helix, E/e: strand, L/l: coil, assigned by DSSP). Uppercase means structurally equivalent positions with CytC. Lowercase means insertions relative to CytC.

From a structure-function perspective, the tridimensional structure of the *Dickeya* CytC toxin, and its backbone dynamics, were determined by NMR spectroscopy. CytC NMR structure is very similar to the crystal structures of the mature monomer Cyt1Aa (3RON.pdb)^19^, of the endogenously cleaved Cyt2Ba monomer (2RCI.pdb)^16^ and the corresponding region of Cyt2Aa (1CBY.pdb)^35^ despite their low sequence identity (~25%; Figures 6 and 7). Unlike *Bt* Cyt toxins, the *Dickeya* CytC was not cleaved to obtain a soluble toxic monomer, and this ability is confirmed by a correlation time τ_c_ indicative of a monomeric state. Cyt1Aa contains an insertion of a β-hairpin between α1 and α2 which is common to all members of the Cyt1 family and is absent from the Cyt2 family and from the Cyt proteins of *D. dadantii*. We can then conclude that CytC is structurally a Cyt2-like protein. This is confirmed by a higher percentage of structural similarity between Cyt2Aa, Cyt2Ba and the "closed" conformation of CytC, 65% than between Cyt1Aa and CytC, 59–60% (Figure 7). The most accepted mechanism by which Cyt proteins damage cell membrane is similar to the one proposed for the volvatoxin A2^36^,^37^ (1VCY.pdb). Firstly, the protein undergoes conformational changes where the two outer α-helical layers swing away from the β-sheet. The β-sheet is then able to bind on the cell membrane and finally oligomerization on the cell membrane forms β-barrel pores. Nevertheless, recent studies^32^ suggested that oligomerisation is a prior step before Cyt1Aa membrane insertion. The NMR structure of CytC is clearly in agreement with the first step of this proposed mechanism as α-helices could have the ability to swing away from the β-sheet in solution. Moreover, this behavior could explain the lack of stability of the protein. Indeed, once in "open" conformation, oligomerization might occur, leading to protein precipitation. The pores, in Cyt1Aa, have been proposed to be formed by three major β-strands (β6–β8) which are structurally conserved in CytC (β2–β4)^32^.

CytC, like other Cyt family members, also has a fold similar to that of the virulence factor Evf (2W3Y.pdb) despite its very low (~15%) sequence identity. In the case of Evf, a palmitate covalently bound by a cysteine is found in a hydrophobic pocket embedded between the β-sheet composed of β3, β5, β6 and β7 strands and α4 and α5 helices (Evf numbering). The structural homology between Cyt proteins and Evf enabled the identification of a putative fatty acid binding site in all Cyt1 and Cyt2 protein between the sheet formed by β4, β6–β8, and helices α3–α5 (Cyt1Aa numbering)^38^. CytC NMR structures also exhibit a quite large hydrophobic pocket defined between the β-sheet and α3 and α4 in the “closed” conformation (models 1 to 10) and of course absent in the “opened” conformation (models 11 to 20) (Figure 5). Moreover the hydrophobic residues delineating the cavity are conserved among the Cyt family members (Figure 6). The presence of this hydrophobic cavity constitute a strong evidence that the common ancestor of Evf and the cytolytic toxins contained a lipid binding site which has been maintained in the two clades, and probably in most members of the Cyt-toxin family, since *Eca*-Evf, *Bth*-Cyt2B and *Dda*-CytC are located at diverse positions of the phylogenetic tree (Figure 2). These data support the importance of this putative lipid-binding cavity for the mechanism of action of Cyt and Cyt-like family members, since hydrophobic interactions would clearly prevail at the membrane binding site(s).

In conclusion, the susceptibility of aphids to *D. dadantii* Cyt endotoxins, and some specificities of the latter such as their non hemolytic properties, may lead to the development of effective strategies for controlling such sucking pests with genetically modified crops expressing the toxins. However, two conditions should concur. (i) Toxins must be expressed in the plant phloem to be accessible to these pests and (ii) more effective toxins should be found or engineered. As example, Chougule and collaborators have improved toxicity of *Bacillus thuringiensis* toxin Cyt2Aa against hemipteran insect pests. Insertion of a 12-amino-acid pea aphid gut-binding peptide by adding to or replacing amino acids in one of three loops of the *Bt* cytolytic toxin, Cyt2Aa, has resulted in enhanced binding and toxicity against both the pea aphid, *Acyrthosiphon pisum*, and the green peach aphid, *Myzus persicae*^25^. The exploration of our results may end up in a new protein family lead for the control of aphids and related insect pests, which include some of the most important pests of global agriculture.

## Methods

### Over-expression of four D. dadantii cyt proteins and ^15^N and ^13^C labeling of CytC

*cytA* was amplified with the oligonucleotides CytA+ (5′-cctgggatccaaattcgacaatattgtcgttc-3′) and CytA- (5′-gccgctcgagggcgagcatggcatttttag-3′), *cytB* was amplified with the oligonucleotides CytB+ (5′-cctgggatccaacaatattgcattgaatccga-3′) and CytB- (5′-gccgctcgagggttgatagatccagtctgcc-3′), *cytC* was amplified with the oligonucleotides CytC+ (5′-cctgggatccaacaatattgcattgaatccga-3′) and CytC- (5′-gccgctcgagggttgatagatccagtctgcc-3′), and *cytD* was amplified with the oligonucleotides CytD+ (5′-cctgggatccgtcagggggtatgctttacagg-3′) and CytD- (gccgctcgagccgctgggacttgggtcgcggc-3′). The amplified DNA were digested with BamHI and XhoI and ligated into pGEX-6p3 plasmid (GE Healthcare) digested with the same enzymes. The pGEX derivatives producing the fusion GST-Cyt proteins were introduced into *E. coli* NM522 strain. Cells were grown in LB medium to OD_600_ 0.8 and induced with 1 mM isopropylthiogalactoside (IPTG) for 3 h.

Labeling of CytC was performed according a method adapted from Marley *et al*^39^. *E. coli* NM522/pGEX-CytC was grown in 4 L of LB medium. When OD_600_ reached 0.8, bacteria were collected by centrifugation and resuspended in 1 L of M63 medium containing 2 g/L ^15^N NH_4_Cl. After 1 hour of growth, 10 mL of a 20% ^13^C labelled glucose solution and 1 mM IPTG were added. Cells were grown overnight and treated as described thereafter.

### Purification of recombinant labeled and unlabeled proteins, and toxin activation

Cells were collected by centrifugation, resuspended in buffer A (50 mM, Tris pH 7.0, 100 mM NaCl, 1 mM EDTA) and broken in a French cell press. Unbroken cells were eliminated by centrifugation. GST-Cyt proteins were bound on Protino Glutathione Agarose 4B (Macherey-Nagel) equilibrated with buffer A, washed several times with the same buffer and the Cyt proteins were liberated by addition of Prescission® protease (GE Healthcare) according to the manufacturer's protocol. The proteins used for bioassay tests were dialyzed extensively against pure water and freeze-dried.

For checking the potential effect of proteolytic cleavage on bioactivity, preliminary hemolysis and insect bioassays with activated/non activated CytC were performed. Purified (desalted, lyophilised) CytC was activated by trypsin (Sigma P-7926) directly in AP3 pH 7.5 insect diet^40^. Toxin was incubated at 500 mg/mL with purified trypsin at 1/25 toxin/protease ratio, for 16 h at 25°C, and proteolysis was arrested by adding 2% v/v fresh 100 mM aqueous PSMF; PMSF decay was left for 4 h before start of assays, and PMSF controls showed that PMSF had no effect on assay in these conditions^18^. SDS-PAGE controls identified toxin purity and main lysis products.

Cyt1A was produced as previously published^18^ from a recombinant *Bacillus thuringiensis* subs. *israelensis* strain.

### Hemolysis assays

Erythrocyte lysis was monitored by hemoglobin release similar to the procedure described previously^41^. Sheep red blood cells diluted at 1% in PBS were incubated with increasing amounts of CytA, CytB or CytC toxins. Moreover, CytC were used with or without trypsin proteolytic activation (see above). After 2 h at room temperature, unbroken cells were removed by centrifugation at 13 000 g for 30 s and the OD_540_ of the supernatant was measured. A positive control was performed by adding 0.1% Triton X100 to the red blood cells, and trypsin-activated Cyt1A^18^ served as an additional positive control.

### Insects and insect assays

The aphid clone used was *Acyrthosiphon pisum* LL01, a long-established alfalfa-collected clone for use in the laboratory, and it was grown on *Vicia faba* (cv. Aquadulce). This genotype has been used in all our previous works on *A. pisum/D. dadantii* interactions and the growth inhibition and survival analyses have been fully described elsewhere^17^,^18^,^40^.

### Statistics

All aphid mortality data were analyzed by a standard survival analysis with the JMP software. Statistical comparisons and confidence calculations were done using the parametric module with a Weibull fit (graphically tested to best fit the aphid survival data).

### Phylogeny

Homologous proteins were recovered in a two-step process: i) Blast searches (either *Blast* or *Delta-Blast*, NCBI) were run using CytA as seed and standard settings, usually E 10^−2^ threshold, against *nr* and *environmental proteins* database (March 2013). All toxins were recovered and redundant *Bacillus thuringiensis* toxins were cleared after a first round of phylogenetic analysis, to keep only one member of each clade of the official *Bt* toxin nomenclature^23^; ii) extended Blast searches (PSI-Blast, NCBI) were also run with all toxins recovered in the first set, as well as toxins that were shown in the literature to belong to the structural family of *B. thuringiensis* Cyt toxins, namely the Evf proteins^20^. This search recovered proteins belonging to three annotation groups: Cyt-like toxins, Evf proteins and volvatoxin-related proteins.

A protein-based phylogeny was built using all recovered proteins in the homology searches, excluding the redundant and one chimeric protein (Ype-Cyt/Q7ARC7, comprising a truncated Cyt-like module). Due to the distant relationship between members of three groups of retrieved homologs, the alignment was built on a structural basis using dialign and an expert-based analysis of the alignment to keep aligned the structural elements of the protein groups (based on the published structures of Cyt, Evf, volvatoxin and the *Dickeya* toxins). The phylogeny was then performed using Seaview^42^ and a ML tree search (WAG model with 4 rate classes, 450 informative sites analyzed; WAG model was chosen after likelihood analysis of the different protein evolution models available in SeaView/ML).

### NMR spectroscopy and structure calculation and dynamics analyses

Samples of 0.3 mM ^15^N, ^13^C labelled CytC protein in 90% H_2_O, 10% D_2_O containing 50 mM Tris (pH 7.0), 100 mM NaCl, 1 mM EDTA and 5 mM DTT were used for NMR spectroscopy. All NMR experiments were performed on a 600 MHz Varian ^UNITY^INOVA spectrometer at 298K. Spectra were processed with NMRPIPE^43^ and analysed with CCPNMR (version 2.1.5)^44^. Backbone and side-chain resonance assignments were obtained from the standard triple resonance experiments^45^. Interproton distances were derived from 3D ^15^N-HSQC-NOESY and ^13^C-HSQC-NOESY datasets obtained at a mixing time of 100 ms. Backbone dihedral angle restraints were determined with DANGLE programs^46^.

Structures were calculated with NOE distances, hydrogen bonds and φ and ψ angles using ARIA2 (version 2.3)^47^. The ARIA2 protocol used simulated annealing with torsion angle and Cartesian space dynamics with the default parameters, including water refinement of the structures. The iterative process was repeated until the assignment of the NOESY spectra was complete. The last run was performed with 500 initial structures out of which 20 were selected on the basis of total energies and restraint violation statistics, to represent the structure of CytC in solution. The figures were prepared with PYMOL. ^15^N R_1_ and R_2_ spectra were acquired with 32 scans per t_1_ point, with a recycle delay of 3.0 s. R_1_ relaxation delays of 10, 50, 100, 200, 300, 400, 500, 600, 700, 800, 1000, 1500, 2000 and 3000 ms were used for data collection. R_2_ relaxation delays of 10, 30, 50, 70, 90, 110, 130, 150, 170, 190, 210, 230, 250, 310, 350 and 410 ms were used for data collection. R_1_ and R_2_ were obtained using a single exponential decay function.

The ^15^N-NOE spectra were collected with a 3 s presaturation period and a 3 s relaxation delay; the reference experiment had an equivalent 6 s delay. The ^1^H-^15^N heteronuclear NOE was calculated from the equation NOE = I_sat_/I_eq_, with I_sat_ and I_eq_ the intensities of a cross peak in the spectra collected with and without presaturation respectively.

### Sequence alignment for structural analyses

Sequence alignments were performed using CLUSTAL OMEGA^48^ and analyzed with JALVIEW^49^ programs.

## Supplementary Material

Supplementary Information Supplementary Figure S1

## Figures and Tables

**Table 1:** NMR constraints and structural statistics

NMR constraints	
Distance restraints	
Total NOE	4522
Unambiguous	4276
Ambiguous	246
Hydrogen bonds	83
Dihedral Angle Restraints	351

Structural Statistics (2 mlw.pdb)	Models 1–10	Models 11–20
Average violations per structure		
NOEs ≥ 0.3 Å	0	0
Hydrogen bonds ≥ 0.5 Å	1	1
Dihedrals ≥ 10°	0	0
Average pairwise rmsd (Å)	0.357	0.329
	0.467	

Ramachadran Analysis	
Most favored region	60.4%
Allowed region	34.2%
Generously allowed	4.0%
Disallowed	1.4%
