# Supplementary material for: New Cyt-like δ-endotoxins from Dickeya dadantii: structure and aphicidal activity
Source: Sci Rep. 2015 Mar 5;5:8791. doi: 10.1038/srep08791 (PMC5390079; doi:10.1038/srep08791)
Supplement: Supplementary Information — Supplementary Figure S1 [file srep08791-s1.doc]

**New Cyt-like δ-endotoxins from *Dickeya dadantii*: structure and aphicidal activity**

Karine Loth^5 ¶^, Denis Costechareyre^1,2,3,4 ¶^, Géraldine Effantin^1,2,3,4^, Yvan Rahbé^1,4,6^, Guy Condemine^1,2,3,4^, Céline Landon^5^ and Pedro da Silva^1,4,6^ **^*^**

1: INSA-Lyon, Villeurbanne F-69621, France.

2: CNRS, UMR5240 MAP, Microbiologie Adaptation et Pathogénie, F-69622, France.

3 : Université Claude Bernard Lyon 1, F-69622, France.

4 : Université de Lyon, F-69000 Lyon, France.

5 : Centre de Biophysique Moléculaire, CNRS UPR 4301, Université d’Orléans, Orléans, F‑45071, France

6: INRA, UMR203 BF2I, *Biologie Fonctionnelle Insecte et Interaction*, F-69621, France.

¶: these authors contributed equally to this work

*****To whom correspondence should be addressed**:** Pedro Da Silva, Laboratoire BF2I, INSA Bâtiment Louis Pasteur, 20 avenue Albert Einstein, 69621Villeurbanne cedex, France, Tel.: +33 4 72 43 83 56; Fax: +33 4 72 43 85 34; E‑mail: [pedro.da-silva@insa-lyon.fr](mailto:pedro.da-silva@insa-lyon.fr)

**Figure S1**:

Coomassie Blue-stained SDS-polyacrylamide gel of Cyt A, B, C proteins.


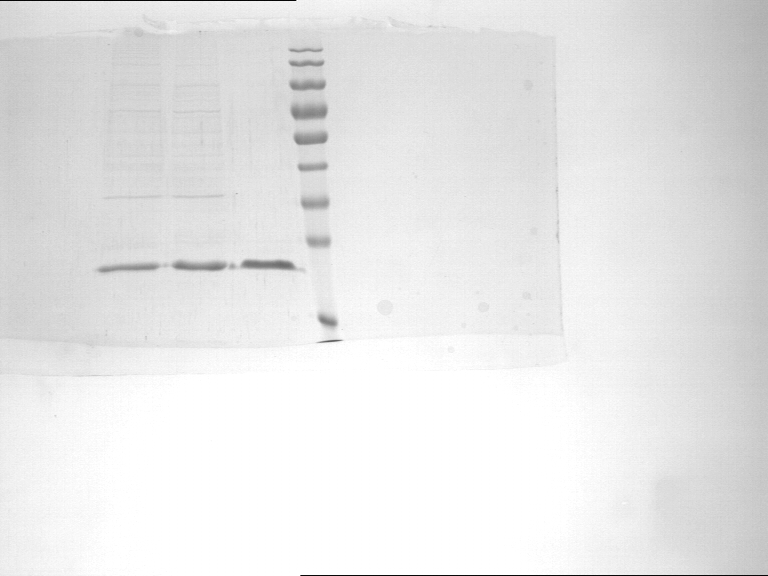


**CytA CytB CytC M**

**kDa**

170

130

100

70

55

40

35

25
